# An experimental study of the physical mechanisms of fluid flow in tight carbonate core samples by binary surfactants

**DOI:** 10.1016/j.heliyon.2025.e42221

**Published:** 2025-02-07

**Authors:** Ayomikun Bello, Anastasia Ivanova, Alexander Rodionov, Tagir Karamov, Andrey Morkovkin, Alexey Cheremisin

**Affiliations:** Center for Petroleum Science and Engineering, Skolkovo Institute of Science and Technology, Skolkovo Innovation Center, 11 Sikorskiy Street, Moscow 143026, Russia

**Keywords:** Adsorption, Wettability, Imbibition, Binary surfactants, Carbonates

## Abstract

Binary surfactants present a promising approach to modifying the petrophysical mechanisms of rock formations to enhance fluid flow, particularly in challenging environments like carbonate rocks. Carbonate rocks exhibit a complex surface charge, which makes it difficult to generalize the use of traditional single surfactants. Hence, the application of binary surfactant systems is proposed as a more effective alternative. This study investigates fluid-rock interactions through adsorption, wettability alteration, and spontaneous imbibition tests. First, static adsorption tests were conducted on eight different surfactant systems to compare the adsorption behaviors of the binary surfactant systems with those of individual surfactants. The results showed a significant influence of the nonionic surfactant with a considerable reduction in adsorption values of 53% and 28% in its anionic-nonionic and cationic-nonionic blends, respectively. Although contact angle measurements taken after aging oil-treated carbonate discs in binary surfactant solutions indicated that wettability was not significantly altered, the binary systems demonstrated the highest efficiency in terms of oil production during spontaneous imbibition tests. Specifically, the zwitterionic-nonionic surfactant system recovered 58% of the initial oil in core samples, compared to 31% and 25% when zwitterionic and nonionic surfactants were used individually. Thus, the use of binary surfactant systems shows great potential for improving oil recovery efficiency, and the findings may have broader implications for optimizing filtration mechanisms in carbonate reservoirs.

## Highlights

• Low adsorption of surfactants ensures maximum interfacial activity in porous media.
• Nonionic surfactant reduced adsorption by 53% with anionic, 28% with cationic surfactants.
• Nonionic surfactant acts as a co-surfactant to boost solubility of the ionic component in a binary system.

## 1 Introduction

Fluid interactions with reservoir rocks influence critical factors such as the recovery of oil and gas, fluid migration, reservoir performance, and geological carbon storage [1], [2], [3]. Therefore, it is important to understand the behavior and mechanisms of fluid interaction in porous media to enhance the efficiency of these processes and accurately forecast their results. Among the diverse range of porous media, carbonate reservoirs constitute a significant portion of global hydrocarbon reserves, and their efficient exploitation is of paramount importance to meet increasing energy demands [4], [5]. However, traditional recovery methods often leave a significant fraction of oil trapped within the pore spaces of carbonate rocks.

There has been significant research conducted on surfactants to investigate their potential to modify interfacial properties and enhance fluid flow within porous media. Surfactants exhibit amphiphilic characteristics, possessing a hydrophobic tail and a hydrophilic head [6], [7]. These enable them to adsorb at the interface between fluids and rocks, thereby reducing interfacial tension and altering wettability. Consequently, this leads to an improved mobilization and displacement of oil. The adsorption behavior and subsequent alteration of wettability depend on a range of factors, including the surfactant structure and concentration, pH, temperature, salinity, and mineralogy of the porous media. The understanding of surfactant behavior and its influence on interactions between fluids and rocks in sandstones has been fairly well established over time [8], [9], [10]. However, the same level of understanding cannot be applied to carbonates, which present a unique set of challenges due to their inconsistent surface charge and complex mineral compositions [11], [12].

Sandstones typically consist of silica and a significant proportion of clay minerals, including kaolinite, chlorite, and montmorillonite. Due to silica's negative charge across a wide pH range, sandstones are generally characterized by a negative charge [13]. Consequently, anionic surfactants are predominantly employed for surfactant flooding in sandstones because of the electrostatic repulsion between the rock and the surfactant, which hinders adsorption. On the contrary, characterizing carbonates is quite complex because their overall surface charge can either be negative or positive, depending on the amount of clay impurities they contain. This variation arises from the presence of minerals such as dolomite, calcite and magnesite, as well as other impurities [14]. Consequently, carbonates exhibit a more complex surface charge.

Several research studies have investigated the behavior of surfactants in various geological formations [2], [11], [14], [15], [16]. These studies have provided evidence that anionic surfactants possess the ability to decrease the interfacial tension between oil and water, modify wettability, and facilitate the recovery of oil in sandstone formations. However, the effectiveness of anionic surfactants in carbonate formations has revealed a greater degree of inconsistency, primarily attributed to the presence of divalent cations and fluctuations in surface charge.

Cationic surfactants exhibit an increased attraction to carbonate minerals and can adsorb to the rock surface, resulting in alterations in wettability and enhanced efficiency in displacing oil [17], [18]. The positive charge associated with cationic surfactants facilitates electrostatic interactions with negatively charged carbonate surfaces, thereby facilitating their adsorption and reducing interfacial tension [11]. However, it should be emphasized that most carbonate formations are characterized with a substantial presence of divalent ions like Ca^2+^ and Mg^2+^. Consequently, surfactants tend to adsorb significantly, forming calcium and magnesium salts that may partition into the oil phase or precipitate. Although divalent cations introduce positive charges to carbonate surfaces, thereby minimizing the adsorption of cationic surfactants, the existence of silica, clay or other impurities within carbonates can incite the adsorption of cationic surfactants [19], [20]. Thus, the complexity of carbonate surface charge renders it challenging to establish a generalized approach for using either cationic or anionic surfactants to reduce surfactant adsorption on carbonate surfaces.

To emphasize, the surface chemistry of carbonates plays a significant role in the adsorption of surfactants. In Ma et al. (2013) [11], the adsorption behavior of cationic and anionic surfactants on carbonates was investigated. Although, the authors used synthetic calcite in their study, which does not accurately represent reservoir conditions, nevertheless, their findings demonstrated that the adsorption of cationic surfactants on calcite is minimal when compared to anionic surfactants, which was attributed to the strong electrostatic repulsion between the cationic surfactant and the positively charged calcium ions on the calcite. Additionally, the results showed significant adsorption of cationic surfactants and negligible adsorption of anionic surfactants on silica, which is in contrast to the outcomes observed with synthetic calcite. Therefore, we can say that if carbonates contain a considerable amount of silica ions, significant adsorption of cationic surfactants may occur.

Ahmadali et al. (1993) [16] conducted similar experiments to compare the adsorption of anionic and cationic surfactants on two pure carbonate minerals, namely calcite and dolomite. The findings, similar to those of Ma et al. (2013) [11], revealed that cationic surfactants exhibited significantly less adsorption on both dolomite and calcite than with anionic surfactants. Furthermore, Ahmadali et al. (1993) [16] noted that the presence of divalent ions such as Ca^2+^ and Mg^2+^ reduced the adsorption of cationic surfactants on carbonates. This was attributed to the fact that divalent ions render the carbonate surface more positively charged, thereby causing the repulsion of cationic surfactant molecules from the interface due to electrostatic interactions.

In a more extensive study, Dehaghani et al. (2020) [15] found synergistic effects in the combination of CTAB and smart water (sea water modified by increasing the concentration of SO${}_{4}^{2-}$) for fluid-rock interaction in carbonates. The authors explained that the decrease in positive charge on the surface of carbonate rocks facilitated the adsorption of CTAB monomers onto the surface. Furthermore, their contact angle and zeta potential tests revealed alterations in the wettability of the rock surface in the presence of CTAB, promoting a shift towards a water-wet state. These findings underscore the inconsistent nature of carbonate surface charge, hindering the generalization of cationic or anionic surfactant applications. Moreover, it is essential to note that surfactant adsorption is not exclusively advantageous, as its effects can be both positive and negative depending on the intended purpose of the process [11]. Surfactant adsorption proves beneficial when the objective is to modify wettability through the adsorption mechanism of surfactant molecules. However, when the presence of surfactant molecules in the aqueous phase is vital for applications such as interfacial tension reduction, surfactant adsorption exhibits a detrimental effect.

Thus, in Kumar and Mandal (2019) [14], the authors used amphoteric surfactants, which possess both positive and negative charges to investigate adsorption on carbonates and sandstones. The findings of the study revealed a greater level of adsorption of the amphoteric surfactant on carbonate samples compared to sandstones. Conversely, the surfactant exhibited enhanced wettability alteration in sandstone samples as opposed to carbonate samples. This phenomenon was attributed to the formation of ion pairs between the amphoteric hydrophilic head and the adsorbed components of crude oil on the surfaces of both sandstones and carbonates. These promising outcomes indicate that surfactant systems that contain both positive and negative charges in their hydrophilic heads can be used in both carbonate and sandstone reservoirs.

Somasundaran and Krishnakumar (1997)[21] studied the adsorption behavior of a binary surfactant system consisting of both non-ionic and ionic surfactants. The authors attributed the significant enhancement of adsorption observed when non-ionic surfactants are combined with ionic surfactants, and vice versa, to the use of the surfactants in mixtures, which compels them to adsorb onto surfaces that individually they would not readily adsorb to [22]. The observed synergistic effect was attributed to the reduction of charge repulsion, thereby promoting a more efficient packing of the ionic surfactant molecules. In the case of non-ionic surfactants, their increased adsorption was attributed to their solubilization within the hydrophobic microdomains formed by the presence of the ionic surfactants.

The investigation of binary surfactant systems presents a relatively new and promising approach to improve fluid-rock interactions and fluid flow in carbonate formations. Binary surfactant mixtures are comprised of two surfactants possessing complementary characteristics, such as anionic-cationic or anionic-nonionic. The combination of surfactants can lead to synergistic effects, enhancing the overall performance in terms of interfacial tension reduction, wettability alteration, and oil recovery. However, despite these advantages, there remains limited understanding of the detailed mechanisms through which these binary systems influence fluid-rock interactions, particularly in complex carbonate formations.

Given the knowledge gaps, this study aims to investigate the fluid-rock behavior of binary surfactant systems in carbonate reservoirs. In our previous work [23], we conducted a fluid-fluid interaction study to characterize synergism and antagonism between surfactants in a binary system. The results were promising as binary surfactant systems demonstrated relatively lower critical micelle concentration (CMC) values compared to single surfactants. This indicates that they could be used at lower concentrations to achieve desired interfacial and recovery results. In this work, we focused on static adsorption, wettability alteration and spontaneous imbibition tests to provide a comprehensive understanding of the fluid-rock interactions involved. The findings from this research will contribute to filling the existing knowledge gap, enabling the formulation of more effective surfactant-based enhanced oil recovery (EOR) for carbonate reservoirs.

## 2 Experimental section

### 2.1 Materials

The materials used in this study represent a carbonate geological formation situated in the Volga-Ural region, Russia. All surfactants used are commercially available. Detailed information regarding each material is provided in the subsequent sections.

#### 2.1.1 Brine

The composition of the formation brine used in this study is described in Table 1. The conductivity and pH were measured at 25 ^∘^C and obtained as 37.86 mS/cm and 7.12, respectively.

**Table 1:** Brine composition.

Salt	NaCl	KCl	CaCl_2_	MgCl_2_	NaHCO_3_	Na_2_SO_4_
Composition, ppm	17211	426	5586	3350	30	71

#### 2.1.2 Surfactants

In this study, we prepared eight surfactant compositions, which included both single surfactants and binary mixtures. These compositions were formulated in the brine described in Table 1, using the fluid-fluid interaction data obtained from our previous work. Critical micelle concentrations (CMC) for these surfactant compositions were determined in our previous work, taking into consideration the relevant salinity and temperature conditions. For this work, each surfactant was used at concentrations 1.5 times higher than its CMC. This approach ensures that the surfactants are used above their CMCs, enhancing their performance and forming stable solutions. The details are given in Table 2.

**Table 2:** Surfactant compositions used in this study.

No.	Composition	Chemical composition	Supplier	Surfactant type	Concentration
**SINGLE SURFACTANTS**					

#1	CTAB	Cetyltrimethylammonium Bromide	Sigma-Aldrich, *Missouri, USA*	Cationic	0.105%
#2	AOS	Alpha Olefin Sulfonate	Stepan, *Wesseling, Germany*	Anionic	0.315%
#3	FARUS	Oxyethylated phenols in monatomic alcohols	Farus JSC, *Moscow, Russia*	Non-ionic	0.480%
#4	BETAINE	Cocamidopropyl betaine	Aminokhim, *Moscow, Russia*	Zwitterionic	0.525%
**BINARY SURFACTANT SYSTEMS**					
#5	AOS + FARUS (1:2)	-	-	-	0.135%
#6	CTAB + FARUS (1:2)	-	-	-	0.060%
#7	CTAB + BETAINE (1:1)	-	-	-	0.075%
#8	FARUS + BETAINE (2:1)	-	-	-	0.120%

#### 2.1.3 Oil

**Figure 1:**
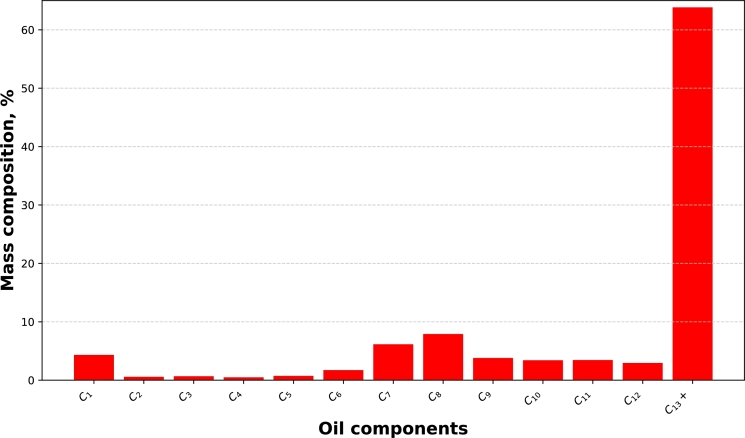
Oil composition.

The crude oil was obtained from the oilfield described earlier in Section 2.1. The density and viscosity of the oil, measured at 41 ^∘^C are 0.8504 g/cm^3^ and 1.71 cP, respectively. The components of the oil as well as its SARA analysis are shown in Fig. 1 and Table 3, respectively.

**Table 3:** SARA Analysis of crude oil sample.

Saturates (%)	Aromatics (%)	Resins (%)	Asphaltenes (%)
37.86	45.34	11.81	4.99

#### 2.1.4 Core samples

Core samples were obtained from the previously described reservoir (Section 2.1) and were used in three distinct sets of experiments. For adsorption tests, the rock samples were crushed, sieved into fine particles of 250 - 300 μm, extracted and then used for the experiment. Regarding wettability tests, the samples were cut into discs, each being 5 mm thick. For the spontaneous imbibition experiments, cylindrical core samples were used. The core samples are carbonates with X-ray diffraction (XRD) analysis showing the following mineralogical composition: Calcite (83.5%), Dolomite (9.5%) and Illite (7%). Their petrophysical properties are presented in Table 4.

**Table 4:** Petrophysical properties of core samples used in this study.

No.	Length (cm)	Diameter (cm)	Porosity (%)	Absolute permeability (mD)	Pore volume, cm^3^
1	3.079	2.975	5.095	0.0124	0.940
2	3.070	2.975	6.524	0.0563	1.264
3	2.690	2.973	7.455	0.0647	1.298
4	2.728	2.976	4.878	0.0097	0.793
5	3.043	2.987	6.933	0.0379	1.393
6	2.388	2.972	7.923	0.0915	1.229
7	3.110	2.984	7.133	0.1857	1.334
8	3.011	2.983	8.731	0.1726	1.753
9	3.061	2.976	8.179	0.0976	1.614

### 2.2 Methodology

#### 2.2.1 Preparation of binary surfactant solutions

Brine solution was firstly prepared by dissolving the appropriate quantity of salt (as in Table 1) in deionized water at ambient temperature. Subsequently, the mixture was subjected to mechanical agitation using a magnetic stirrer at a controlled speed for 3 hours to ensure complete dissolution. Next, the selected surfactants, based on the concentrations and mass ratio in Table 2, were carefully measured using a calibrated analytical balance and added to the brine solution to form binary surfactant systems, and the solution was stirred for an additional hour. The surfactants that were purchased in their liquid form were added to brine solution by recalculating the required volume using the following dilution formula:

(1) $$\;C_{1}\;V_{1}=C_{2}\;V_{2}$$

where: $C_{1}$ = initial concentration of solution, %; $V_{1}$ = initial volume of solution, ml; $C_{2}$ = final concentration of solution, %; $V_{2}$ = final volume of solution, ml.

#### 2.2.2 Static adsorption

Static adsorption test was conducted using crushed carbonate rock samples (SEM image in Supplementary Information Document) and surfactant compositions in brine. Initially, the samples were crushed into particles of 250 - 300 μm. Subsequently, the resulting powder was purified with chloroform (CHCl_3_) using a Soxhlet extractor until the chloroform exhibited visual transparency. The rock powder was then subjected to oven drying to eliminate any remaining chloroform residue. This methodology for preparing the rock powder prior to adsorption experiments is highly effective, as it ensures that residual hydrocarbons from core samples do not interfere with subsequent tests.

To determine the concentration of each surfactant composition in the aqueous media after adsorption onto the carbonate rock surface, interfacial tension (IFT) data was utilized. This technique involves measuring the IFT of surfactant solutions after adsorption and subsequently calculating their concentrations in the solution based on the dependence between IFT and known surfactant concentrations. Calibration curves for each surfactant composition were established by measuring the IFT between the surfactant solution and oil using a spinning drop tensiometer (KRUSS, Germany) prior to conducting the adsorption tests. This methodology has been previously described in our earlier work [23]. As the concentration of surfactant in a chemical composition typically exceeds the critical micelle concentration (CMC), a range of concentrations in the post-CMC region was selected. Calibration curves were then constructed for each surfactant composition, and the corresponding equation from each calibration curve was used to determine the unknown concentration of each surfactant composition after adsorption.

For the adsorption experiment, 5 g of rock powder was accurately weighed, and 15 ml of surfactant solution was added to achieve a consistent rock-to-surfactant solution ratio of 1:3 across all experiments. The experiments were conducted in specialized flat-conical flasks, which facilitate maximum contact between the rock and the solution. The flasks were tightly sealed to prevent evaporation or contamination, manually agitated, placed in a preheated oven at 41 ^∘^C, and left undisturbed to allow for adsorption to take place. After 24 hours, the solution was centrifuged at 2000 rpm for 1 hour to separate the rock particles, and the resulting supernatant was carefully collected for further analysis. The concentration of surfactant remaining in the aqueous phase after rock adsorption was determined, and the adsorption value was calculated using the following formula.

(2) $$\;\Gamma =\frac{C_{b}-C_{a}}{m_{\text{rock}}}\cdot V_{\text{surfactant}}$$

where: Γ = adsorption value, mg/g; $C_{b}$ = concentration of surfactant before adsorption, mg; $C_{a}$ = concentration of surfactant after adsorption, mg; $m_{\text{rock}}$ = mass of rock sample, g; $V_{\text{surfactant}}$ = volume of surfactant solution, ml.

#### 2.2.3 Zeta potential, pH and conductivity measurements

After adsorption, the resulting supernatant was analyzed to determine its zeta potential, pH, and conductivity. The zeta potential was determined using a Zetasizer Nano (Malvern Instruments, UK). This instrument measures the electrophoretic mobility of particles in a liquid medium, enabling the calculation of the zeta potential based on the Smoluchowski equation. To perform the measurement, the sample was carefully transferred into a clean and uncontaminated cuvette using a pipette. Subsequently, the cuvette was carefully placed into the sample chamber. Then, the sample was allowed to equilibrate at 41 ^∘^C for 120 seconds which is sufficient to ensure thermal stability. Each sample was measured three times, with 100 runs conducted for each measurement, and the average value was recorded.

The pH and conductivity of the samples were determined using a Conductimeter (Mettler Toledo, USA). Initially, the instrument was calibrated using standard buffer solutions with known pH and conductivity values. Following calibration, the electrodes were rinsed with deionized water to remove any potential contaminants. Subsequently, the electrodes were immersed in the samples, ensuring complete submersion without making contact with the sides or bottom of the container. Upon stabilization, the instrument displays the pH and conductivity values of the solution. Each sample was measured three times and the average value was recorded.

#### 2.2.4 Wettability

The wettability was assessed on the macro level through contact angle measurements, using the sessile drop method on the Kruss drop shape analyzer 30S. The technique involves measuring the contact angle (on both sides) between the rock surface and the wetting liquid. The resulting images were processed using Kruss Advance software. With the help of this program, the shape of the droplet can be set along the contour and then the contact line can be determined.

In this work, carbonate samples were cut into thin slices and placed on the measuring stand of the device using tweezers. Deionized water was used as a wetting fluid. The contact angle between the surface of the carbonate sample and deionized water was calculated as the average value from a sample of at least 20 contour measurements. Measurements were taken at different points of the sample (at least 10 points).

Three sets of contact angle measurements were carried out:

• Carbonates are known to be predominantly oil-wet or mix-wet so the initial wettability of the samples were measured “as is”.
• After the slices were cleaned with toluene and dried, they were placed in jars with oil, and then in oven preheated to 41 ^∘^C for 14 days. Next, each sample was washed with toluene to remove extra oil and dried at room temperature.
• The last set of measurements involves treatment with surfactants, where the oil-wet discs were soaked in surfactant solutions for 48 hours at 41^∘^C.

In all cases, the experimental conditions for the measurements were the same.

#### 2.2.5 Spontaneous imbibition

**Figure 2:**
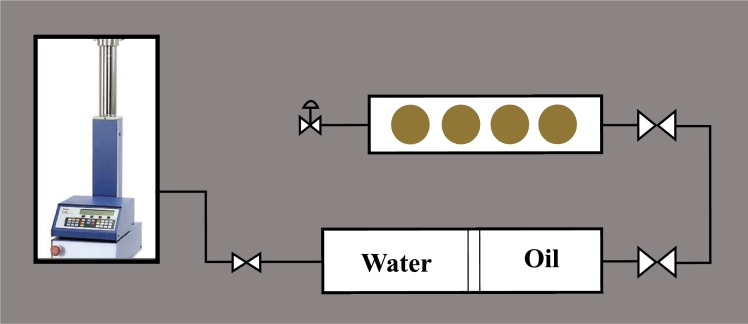
Experimental setup for oil saturation of core samples *(The yellow circles represent the core samples loaded into the pressure cell)*.

Spontaneous imbibition experiments were conducted using Amott cells (Vinci Technologies, France) to investigate the imbibition behavior of core samples in surfactant solutions. Prior to the experiments, the core samples were dried in an oven at 60^∘^C to remove any residual moisture, vacuumed and saturated with oil following the diagram below (Fig. 2). Oil saturation took place in high pressure cylinders at 20 MPa for 12 hours.

The oil-saturated core samples were then placed in the Amott cell, ensuring a tight seal to prevent any fluid leakage. The surfactant solutions were filled into the Amott cell through the inlet tube, displacing the air inside. The cell was then placed in an oven already preheated to 41 ^∘^C to maintain a constant temperature. The process was monitored over 7 days, and the volume of oil produced was recorded at regular intervals.

## 3 Results and discussion

### 3.1 Synergism between binary surfactants for static adsorption

Adsorption on carbonate rocks presents a complex scenario, mainly due to the surface charge arising from impurities and the physicochemical properties of the surrounding aqueous medium. To assess this phenomenon, a static adsorption test was conducted, focusing on the eight surfactant compositions mentioned earlier in this study (Table 2). The primary objective was to compare the adsorption behavior of the binary surfactant mixtures with that of their individual surfactants, aiming to ascertain the synergistic interactions between them. In our previous work, we have stated that synergistic interaction is said to occur when the combined effect of two surfactants yields superior results compared to their individual usage. The obtained results are illustrated in the figure below.

Carbonates are generally known to possess a positive surface net charge [17], [24], which leads to the expectation that cationic surfactants would result in lower adsorption values compared to anionic surfactants. This was the case in Peng and Nguyen (2020)[25] where this was attributed to the electrostatic attraction between the negatively charged head groups of anionic surfactant molecules and the positively charged carbonate rock surface. In contrast, electrostatic repulsion is expected in cationic surfactants such as CTAB, leading to lower adsorption.

**Figure 3:**
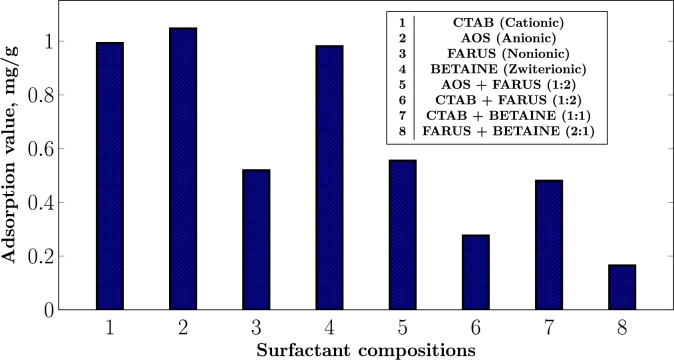
Adsorption capacity of single and binary surfactant systems.

However, as seen in Fig. 3, both ionic and zwitterionic surfactants had almost similar adsorption on the rock surface. This could be attributed to the presence of carbonate ions (CO${}_{3}^{2-}$) arising from the dissociation of carbonate salts in water, as well as the presence of clay minerals such as illite, in the rock samples. As measured, the zeta potential and pH of the carbonate used in formation brine are -4.69 mV and 7.12, respectively. These indicate a negative surface net charge and an almost neutral physicochemical environment.

**Figure 4:**
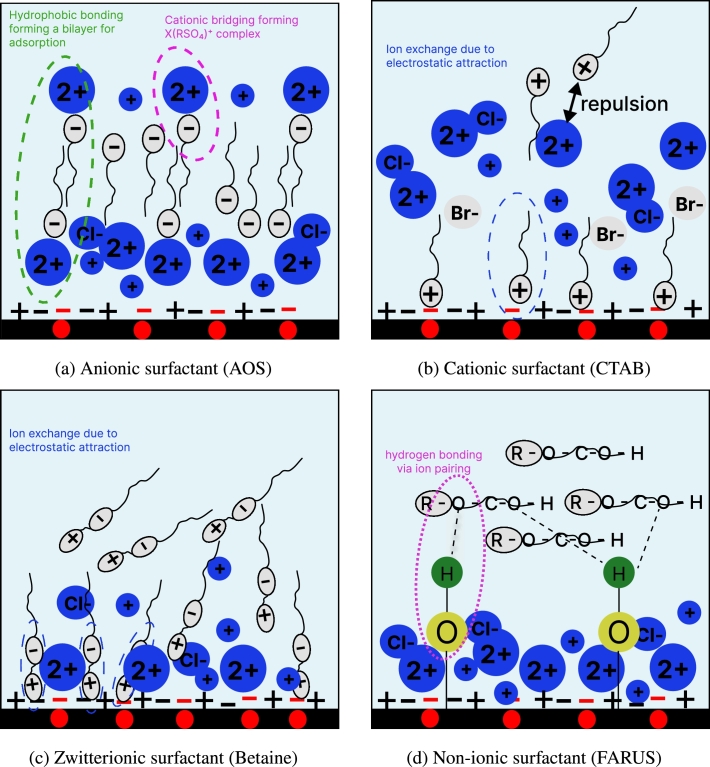
Hypothesized adsorption mechanisms of the single surfactants investigated in this work. *Red spots on the rock surface characterize the presence of negative binding sites due to the presence of illite. Blue charges are from aqueous ions. Grey charges are from surfactant molecules.*

This alteration of surface net charge could be attributed to the presence of divalent cations in the formation brine used, which accounted for 70% of the ions present in the brine. As schematically illustrated in Fig. 4a, divalent ions, such as Ca^2+^ are capable of acting as ionic bridges between anionic surfactants and negatively charged surfaces, thereby favoring anionic surfactant adsorption.

A similar result was found in Ma et al. (2013) [11] where the authors mentioned that anionic surfactants could as well adsorb in carbonates irrespective of the surface charge if the formation water contains divalent cations (Ca^2+^ and Mg^2+^) which can form bridging complexes between the surfactants and the carbonate rock surface. Derkani et al. (2019) [26] explained that the presence of divalent ions in carbonate surfaces facilitates the stabilization of the adsorbed surfactant layer and leads to a reduction in interfacial tension. Furthermore, divalent cations are capable of acting as ionic bridges between anionic surfactants and negatively charged surfaces, thereby favoring anionic surfactant adsorption. The higher the concentration of divalent cations, the more cationic bridges are formed. Although there might still be some anionic surfactant molecules adsorbing directly to the positively-charged binding sites on rock surface, this type of adsorption will be limited due to the concurrent presence of divalent ions distributed across the rock surface, which function as “sacrificial agents” and engage in competitive interactions for binding sites alongside the anionic surfactant molecules.

In the case of cationic surfactant (CTAB), adsorption can be attributed to the ion exchange mechanism which involves the replacement of the surface bound ions with the CTAB molecules (Fig. 4b). Since the net surface charge of the carbonate is negative, it can attract positively charged ions through electrostatic attraction. As CTAB molecules approach the carbonate surface and their positively charged head groups interact with the negatively charged surface, it causes the release of carbonate ions from the surface, which leads to the adsorption of CTAB, molecules onto the carbonate surface (Fig. 4b). This is the same for the zwitterionic surfactant (Fig. 4c) Notably, several works [27], [28], [29], [30] have noted that, in order for cationic surfactants to reduce adsorption on positively charged carbonates, it is important that the mineral surface of the carbonate must be in its pure form, because the presence of impurities such as silica or clay may result in significant adsorption. In fact, Jarrahian et al. (2012) [29] stated that the presence of a little amount of silica and clay may lead to substantial alteration of the carbonate's zeta potential, which is evident in our work.

The adsorption of nonionic surfactant can be attributed to hydrogen bonding. The hydrophilic head of the surfactant contains a polar group, known as ether (-O-) moiety which can form hydrogen bonds with the carbonate surface (Fig. 4d). The key to the adsorption mechanism here lies in the ability of the hydrophilic head of the nonionic surfactant to form hydrogen bonds with the negatively charged carbonate surface. These hydrogen bonds are electrostatic in nature and provide a driving force for the surfactant molecules to adsorb onto the surface. The oxygen atoms in the hydrophilic head of the surfactant act as hydrogen bond donors, while the negatively charged carbonate surface acts as a hydrogen bond acceptor, creating a favorable interaction. However, in carbonate minerals, less hydroxyl groups are present compared to minerals rich in silicate compounds like sandstones, which typically have more available hydroxyl groups [31]. This difference in surface chemistry affects the adsorption of non-ionic surfactants. Since these surfactants lack a charge and are generally non-polar, they find it challenging to form strong interactions with the carbonate mineral surface. As a result, the adsorption of non-ionic surfactants on carbonate minerals is severely limited.

**Figure 5:**
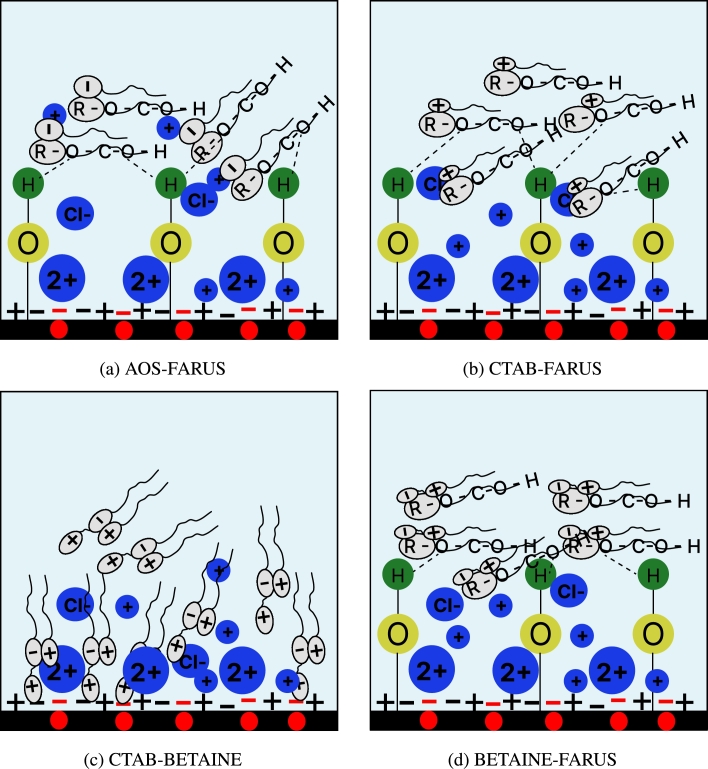
Hypothesized adsorption mechanisms of the binary surfactant systems investigated in this work. *Red spots on the rock surface characterize the presence of negative binding sites due to the presence of illite. Blue charges are from aqueous ions. Grey charges are from surfactant molecules.*

Regarding the binary surfactant mixtures, it is evident from Fig. 3 that their adsorption values are generally lower than those of their corresponding single surfactants, suggesting the presence of synergistic effects. It is important to note that, in some cases, the binary mixtures exhibit both charged groups within the same molecule which leads to a balance of electrostatic interactions, making the adsorption mechanism more complex, for example CTAB-BETAINE (Fig. 5c). Nevertheless, we believe that an important component of the binary mixture is the non-ionic surfactant, as it significantly influences the adsorption behavior of the binary mixtures, for example, the results in the anionic-nonionic and cationic-nonionic binary surfactant systems reveal a substantial influence of the nonionic surfactant, leading to a considerable reduction in adsorption values of about 53% and 28%, compared to their individual surfactants (Fig. 3).

In Bello et al. (2023) [23], we demonstrated that the non-ionic surfactant in a binary surfactant systems acts as a co-surfactant. This is a mechanism for binary surfactant systems, where one of the surfactants is nonionic and can act as a co-solvent or a co-surfactant for the other ionic surfactant. This can increase the solubility of the ionic surfactant in the aqueous phase, reducing its tendency to adsorb on the rock surface. Our results align with those obtained in Das et al. (2020) [32], where the authors studied the adsorption of mixed nonionic and anionic surfactants systems on carbonates. Their results showed that the addition of anionic surfactant as a co-surfactant to the nonionic-anionic surfactant system decreases static adsorption. The authors mentioned that the presence of a charged sulfonate group delays phase separation and increases the cloud point of the nonionic surfactants in the binary system. In all their experiments, the adsorption isotherms of the binary surfactant systems showed that the nonionic surfactant decreased the adsorption of the anionic component in the system. The implication of this that the lower adsorption of binary mixtures indicates that a lesser amount of surfactant is required to achieve the same level of adsorption as single surfactants [10], [33]. Consequently, this implies reduced surfactant consumption, potentially lowering the overall cost of the EOR process.

In addition, competitive adsorption occurs in most of the cases, as shown in Fig. 5. Specifically, we observe that the nonionic surfactant can compete with other ionic surfactants for the adsorption sites on the rock surface. This could be attributed to the fact that the concentration of the non-ionic component in the binary mixtures is higher than other components and, its adsorption capacity, individually, is low. As a result, it can displace or prevent the adsorption of the ionic surfactant, which positively affects and reduces the adsorption capacity of the binary mixture.

### 3.2 Physicochemical evaluations

**Figure 6:**
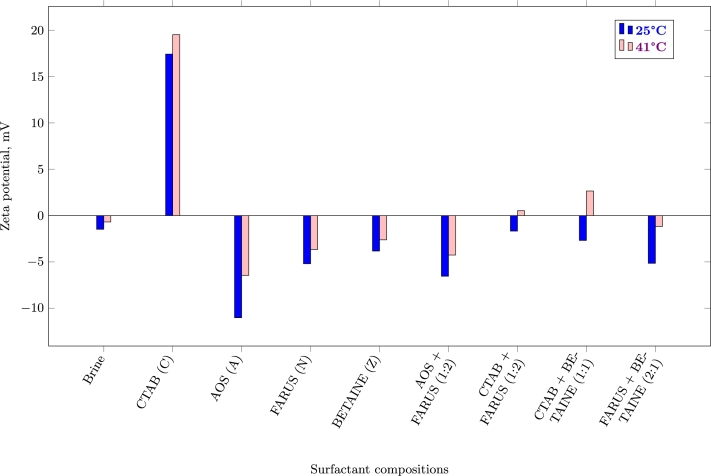
Zeta potential of supernatants after adsorption *(C = Cationic; A = Anionic; N = Nonionic; Z = Zwitterionic)*.

Zeta potential is a critical parameter that characterizes the electrical charge of colloidal particles, including surfaces of solid particles [34]. It reflects the magnitude of the electrostatic attraction or repulsion between charged particles in a colloidal system, which is crucial in understanding the adsorption behavior of surfactants on rock surfaces. A positive zeta potential indicates a net positive charge at the shear plane, while a negative zeta potential corresponds to a net negative charge. A zeta potential close to zero indicates a neutral surface charge [26]. In our study, zeta potential of supernatants were measured after adsorption. The result can be seen in Fig. 6.

In the absence of surfactants, the zeta potential value in brine was -4.69 mV. This value indicates a slightly negatively charged surface. As mentioned in the previous subsection, this is attributed to the presence of carbonate ions (CO${}_{3}^{2-}$) arising from the dissociation of carbonate salts in water, and also the presence of illite which is a clay mineral.

Cationic surfactant, CTAB, exhibited a positive zeta potential of 19.53 mV after adsorption. The positive charge on the surfactant can be attributed to the presence of quaternary ammonium groups in its chemical structure. The high positive zeta potential of CTAB-treated supernatant implies an efficient adsorption on the carbonate surface. The mechanisms for this are explained in detail in previous section. Non-ionic surfactant, FARUS, displayed a slightly negative zeta potential of -3.66 mV. Being a non-ionic surfactant, FARUS lacks electrical charge in its structure. However, the presence of polar functional groups allows for weak interactions with the carbonate rock surface [20], [35]. The modest negative zeta potential suggests a limited ability of FARUS to adsorb on the rock surface, mainly relying on weak hydrogen bonding. In the case of the binary surfactants, we see a significant deviation from their individual surfactants. Cationic and non-ionic surfactant binary mixture was 0.13 mV. This is a significant reduction, especially on the part of the cationic surfactant alone, which suggests an efficient synergism in the reduction of surfactant adsorption on the carbonates.

It is also important to highlight the increase in zeta potential with an increase in temperature. The observed increase in zeta potential with the rise in temperature can be attributed to several underlying processes occurring at the solid-liquid interface during adsorption. El-Mofty et al. (2021) [36] mentioned that as the temperature of the system increases, the mobility of ions in the supernatants also rises. Thus, the thermal energy provided to the system enables ions to move more freely, leading to enhanced diffusion and migration of charged species towards the rock surface. Consequently, the concentration of charged ions near the rock interface increases, resulting in a higher surface charge, i.e., an increase in zeta potential.

We believe that temperature can also influence the degree of ionization of functional groups present on both the surfactant molecules and the carbonate rock surface. Many surfactants possess polar head groups, which can be deprotonated and become charged under certain conditions. Higher temperatures can promote the ionization of these functional groups, leading to a greater number of charged surfactant molecules in the supernatant. As these charged surfactant molecules adsorb onto the carbonate rock surface, they contribute to the overall increase in zeta potential.

**Figure 7:**
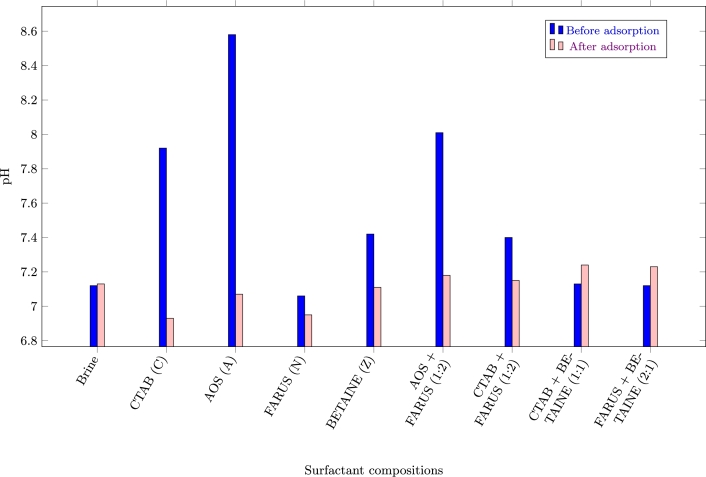
pH values of supernatants after adsorption *(C = Cationic; A = Anionic; N = Nonionic; Z = Zwitterionic)*.

Similar results were obtained for pH (Fig. 7), as we generally see a reduction in the alkalinity (increase in acidity) of the surfactant compositions that adsorbed more onto the carbonate surface. This could be as a result of ion exchange between the carbonates and the surfactants, which is a dominant mechanism of surfactant adsorption [33]. Carbonates are composed mainly of calcium carbonate (CaCO_3_), with carbonate ions (CO${}_{3}^{2-}$) in their structure. So when surfactant molecules, which typically contain charged functional groups (e.g., carboxylates, sulfonates), adsorb onto the carbonate surface, they may undergo ion exchange reactions with carbonate ions. During this process, carbonate ions may be displaced by the surfactant ions, leading to the release of hydroxide ions (OH^−^) and the formation of more acidic species [37].

### 3.3 Wettability evaluation

**Figure 8:**
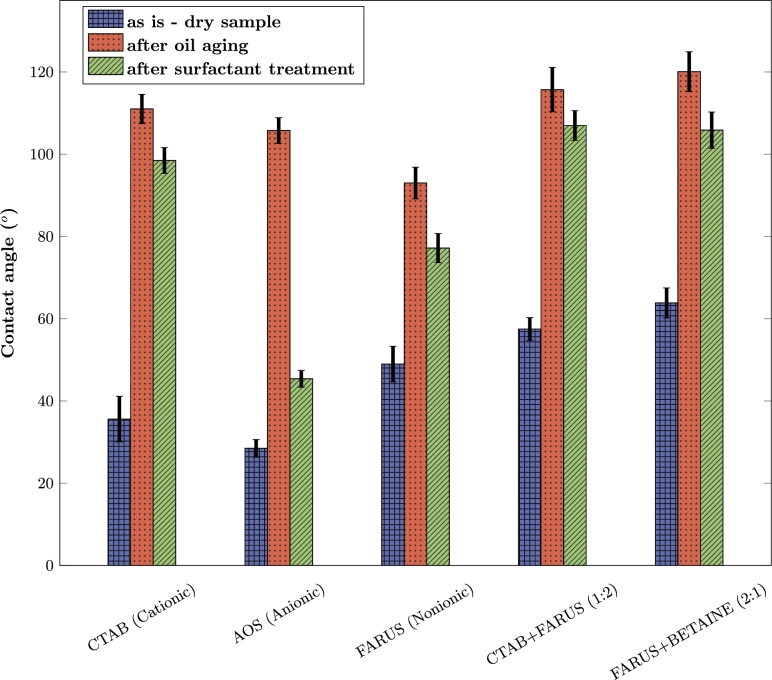
Contact angles of the surfactant systems for wettability evaluation.

The wettability alteration effects were investigated through a series of experiments involving contact angle measurements. The results can be seen in Fig. 8.

As it can be seen, the rock surfaces are originally water wet with contact angles lower than 90^∘^. After aging with oil, a transition to oil wet was observed as the contact angles grew beyond 90^∘^ ranging from 92^∘^ to 120^∘^.

The results in Fig. 8 show a significant wettability alteration in the case of the anionic surfactant, AOS, reducing the contact angle to 35.38^∘^, which highlights its efficiency in reversing the wettability towards a more water-wet condition. This outcome affirms our explanation of the adsorption behavior explained in the previous sub-section (Fig. 3). Although, cationic surfactants are more effective for wettability alteration of carbonate rocks, this only applies to carbonates with a positive surface net charge [29], [30]. CTAB exhibited a contact angle of 95.47^∘^, indicating that the wettability state was still maintained. As a cationic surfactant, CTAB would be expected to strongly adsorb onto negatively charged rock surfaces due to electrostatic attraction, as shown in Fig. 3. This adsorption should, in theory, alter the wettability of the rock surface. We believe that this unusual result could be because the adsorption of CTAB onto the rock surface may have reached a saturation point where further adsorption does not significantly impact the wettability. CTAB molecules could be forming a layer on the surface that does not promote a strong enough alteration in wettability, or the surfactant molecules may align in such a way that their hydrophobic tails are still exposed, limiting the effect on the water-oil-rock interaction.

It is interesting to note that in the static adsorption tests, both CTAB and AOS exhibited similar high adsorption values (Fig. 3). However, wettability tests are quite contrasting. As expected, AOS showed a high wettability alteration capacity from which we can deduce that its mechanism for wettability alteration is its high adsorption value on the carbonate rock due to its electrostatic attraction of charges [38]. CTAB, on the other hand, also had a high adsorption value yet it did not induce a significant wettability alteration (Fig. 3). This suggests that while the surfactant was present on the rock surface, it may not have been effective in altering the wettability significantly. This emphasizes that adsorption alone may not necessarily correlate with wettability alteration and underscores the importance of understanding the underlying mechanisms involved in surfactant-rock interactions.

Interestingly, the binary surfactant solutions used in this study did not significantly change the wettability of the core samples. While FARUS alone altered wettability to hydrophobic, when combined with other surfactants in binary solutions, the wettability of the carbonate samples was maintained. This suggests that the combination of CTAB and FARUS did not synergistically enhance wettability alteration. On a positive side, the poor wettability alteration and low adsorption values of binary surfactant mixtures suggest that the surfactant molecules will be maximally utilized in the porous medium, thereby reducing costs.

### 3.4 Spontaneous imbibition of carbonate rock samples in different surfactant systems

The effects of the surfactant compositions were compared by performing spontaneous imbibition on the carbonate core samples. The specifications of the core plugs used are earlier described in Table 4. At first, a control experiment was conducted with brine only. Oil production was recorded at specified intervals multiple times a day for 1 week until there was no significant oil production from the oil samples. The figure below shows the cumulative production curves.

**Figure 9:**
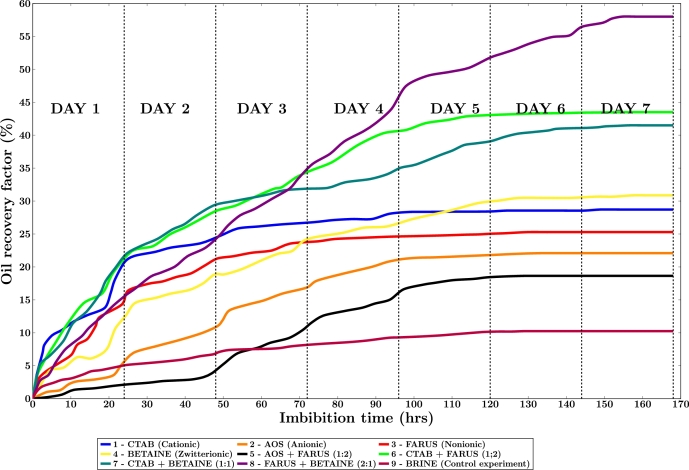
Oil production curves during spontaneous imbibition.

From Fig. 9, we can see that the use of brine alone was not effective in producing significant oil recovery. Brine imbibition resulted in a modest recovery factor of only 17.18% of the original oil in place (OOIP). However, compared to brine imbibition, surfactant-assisted imbibition has substantially larger recovery factors. Nonionic-zwitterionic binary surfactant system showed the highest oil recovery of 58% OOIP, while anionic-nonionic shows the lowest recovery factor (22%). Generally, it can be seen that oil production under spontaneous imbibition with binary surfactants is significantly higher than that obtained with single surfactants.

The poor recovery of AOS in comparison with CTAB can be attributed to their high adsorption on the carbonate surface. Daryasafar et al. (2018) [39] asserted that high adsorption of surfactants can be detrimental to oil recovery. The conditions of the aqueous solution and the rock surface provide a favorable medium for the affinity of the surfactant molecules for the carbonate rock surface. Thus, when these surfactants adsorb onto the rock surface, the availability of the molecules in the bulk solution to interact and mobilize oil is reduced. Consequently, this leads to reduced oil recovery as the surfactant is immobilized on the rock, limiting its effectiveness in reducing interfacial tension and enhancing oil mobility.

This poor oil recovery is more pronounced in AOS because it preferentially altered the wettability of the rock towards water-wet conditions. This makes it more challenging for the surfactant to displace oil from oil-wet regions, which results in poor oil recovery. CTAB on the other hand was able to maintain the oil-wetness of the rock surface.

To further confirm their effectiveness with the binary surfactants, let us consider the binary surfactant mixtures of anionic-nonionic and cationic-nonionic. While the combination of AOS and FARUS did not seem to help the oil recovery that much with a recovery factor of 18.6%, the combination of CTAB and FARUS seems to be more synergistic as we see a low-adsorbing surfactant and good wettability maintaining surfactant, FARUS, producing a recovery factor of 43.5%.

**Figure 10:**
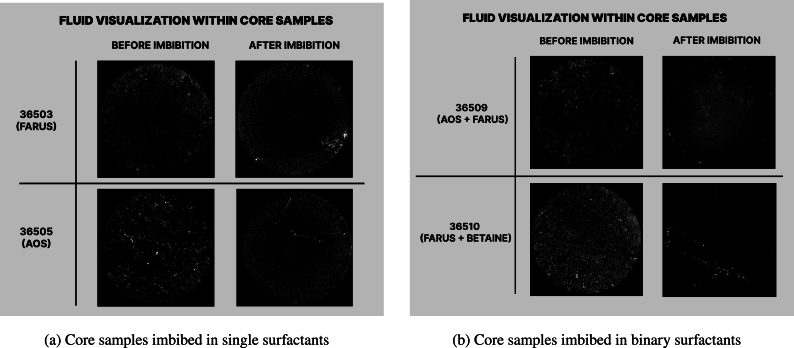
Visualization of fluids within the core samples before and after imbibition experiments *(Bright spots represent oil)*.

To gain a clearer understanding of the fluid distributions within the core samples, a subtraction technique was applied to the CT scan images of the core samples before and after imbibition. (Fig. 10).

Bright spots in the images correspond to the filled pores, indicating the presence of oil. The intensity of brightness directly correlates with the degree of saturation, where brighter regions indicate higher oil saturation. The images appear to corroborate the experimental results of the imbibition process, suggesting a more efficient recovery in the case of the binary surfactants, as we see less bright images after spontaneous imbibition, which suggest low oil saturation.

## 4 Conclusions

In our work, we focused on binary surfactant systems and their interactions with carbonate rock, conducting various laboratory experiments. On the basis of the results of our study, the following several conclusions can be drawn:

• Both ionic surfactants exhibited similarly high adsorption values, but while AOS adsorption was dominated by cationic bridging, CTAB adsorption primarily occurred through ion exchange, where CTAB molecules replaced surface-bound ions.
• Ionic-nonionic binary surfactant systems reveal significant influence of the nonionic surfactant where we see a considerable reduction of around 53% and 28% respectively compared to their individual surfactants.
• The physicochemical evaluations confirmed that the efficient synergism between binary surfactant systems reduced surfactant adsorption in carbonate rocks, as evidenced by decreased zeta potential and pH values compared to their individual surfactants.
• Binary surfactant systems did not significantly change wettability. This could be beneficial as it implies that the surfactant molecules are not adsorbed to the rock surface within the porous medium. Instead, they are utilized to their maximum potential within the porous medium.
• The spontaneous imbibition results showed a notable difference within the ionic-nonionic system. Specifically, when anionic and nonionic surfactants were combined, the recovery factor was 18.6%, whereas in the case of cationic-nonionic, the recovery factor was 43.5%. The highest oil recovery factor of 58% was shown in the zwitterionic-nonionic surfactant system.
• Significant changes in wettability and high adsorption values do not always reflect high oil recovery factors.

The results of this study demonstrate that if carefully screened and combined, binary surfactants can effectively reduce surfactant adsorption, maintain rock wettability and significantly reduce interfacial tension in carbonate rocks, with the aim of enhancing oil recovery. While these findings highlight the potential of binary surfactant solutions to improve fluid flow and alter the physical properties of tight carbonates, several limitations exist. The experiments were conducted at a specific target temperature, so it would be valuable to assess their performance at higher temperatures typical of carbonate reservoirs. Additionally, spontaneous imbibition tests were carried out on different core samples. Although these samples had similar petrophysical properties, it is still difficult to make direct comparisons. Therefore, methods enabling more explicit direct comparisons are recommended for future studies.

## CRediT authorship contribution statement

**Ayomikun Bello:** Writing – review & editing, Writing – original draft, Visualization, Validation, Methodology, Investigation, Formal analysis, Data curation, Conceptualization. **Anastasia Ivanova:** Writing – review & editing, Visualization, Supervision, Methodology, Investigation, Formal analysis. **Alexander Rodionov:** Writing – review & editing, Supervision, Investigation, Formal analysis, Data curation. **Tagir Karamov:** Writing – review & editing, Resources, Investigation, Formal analysis. **Andrey Morkovkin:** Writing – review & editing, Visualization, Resources, Methodology, Investigation. **Alexey Cheremisin:** Writing – review & editing, Visualization, Supervision, Resources, Project administration, Methodology, Funding acquisition, Formal analysis, Data curation.

## Declaration of Competing Interest

The authors declare that they have no known competing financial interests or personal relationships that could have appeared to influence the work reported in this paper.

## Data Availability

The datasets used and/or analyzed during the current study available from the corresponding author on reasonable request.

## Appendix A Supplementary material

MMC Supplementary Information Document. MMC
