# Supplementary material for: An experimental study of the physical mechanisms of fluid flow in tight carbonate core samples by binary surfactants
Source: Heliyon. 2025 Feb 7;11(4):e42221. doi: 10.1016/j.heliyon.2025.e42221 (PMC11867284; doi:10.1016/j.heliyon.2025.e42221)
Supplement: MMC — Supplementary Information Document. [file mmc1.pdf]

Supplementary Information Document for: An Experimental Study of the Physical Mechanisms of Fluid Flow in Tight Carbonate Core Samples by Binary Surfactants

Ayomikun Bello[a], Anastasia Ivanova[a], Alexander Rodionov[a], Tagir Karamov[a], Andrey Morkovkin[a], Alexey Cheremisin[a]

[a] *Center for Petroleum Science and Engineering, Skolkovo Institute of Science and Technology, Skolkovo Innovation Center, 11 Sikorski Street,, Moscow, 143026, Russia*

Abstract

Binary surfactants present a promising approach to modifying the petrophysical mechanisms of rock formations to enhance fluid flow, particularly in challenging environments like carbonate rocks. Carbonate rocks exhibit a complex surface charge, which makes it difficult to generalize the use of traditional single surfactants. Hence, the application of binary surfactant systems is proposed as a more effective alternative. This study investigates fluid-rock interactions through adsorption, wettability alteration, and spontaneous imbibition tests. First, static adsorption tests were conducted on eight different surfactant systems to compare the adsorption behaviors of the binary surfactant systems with those of individual surfactants. The results showed a significant influence of the nonionic surfactant with a considerable reduction in adsorption values of 53% and 28% in its anionic-nonionic and cationic-nonionic blends, respectively. Although contact angle measurements taken after aging oil-treated carbonate discs in binary surfactant solutions indicated that wettability was not significantly altered, the binary systems demonstrated the highest efficiency in terms of oil production during spontaneous imbibition tests. Specifically, the zwitterionic-nonionic surfactant system recovered 58% of the initial oil in core samples, compared to 31% and 25% when zwitterionic and nonionic surfactants were used individually. Thus, the use of binary surfactant systems shows great potential for improving oil recovery efficiency, and the findings may have broader implications for optimizing filtration mechanisms in carbonate reservoirs.

Keywords: , Adsorption, Wettability, Imbibition, Binary surfactants, Carbonates

1. SEM image of crushed sample for adsorption test

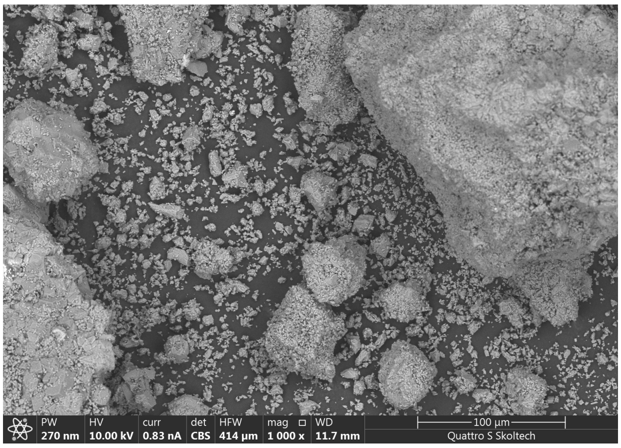

Fig. S1: SEM Image of rock sample used for adsorption test

2. Computed tomography scan of core samples

In order to understand the mechanisms involved on a pore scale level, computed tomography scans were made to take images of selected experiments before oil saturation, after oil saturation, and after the spontaneous imbibition experiments. The figure below shows the results.

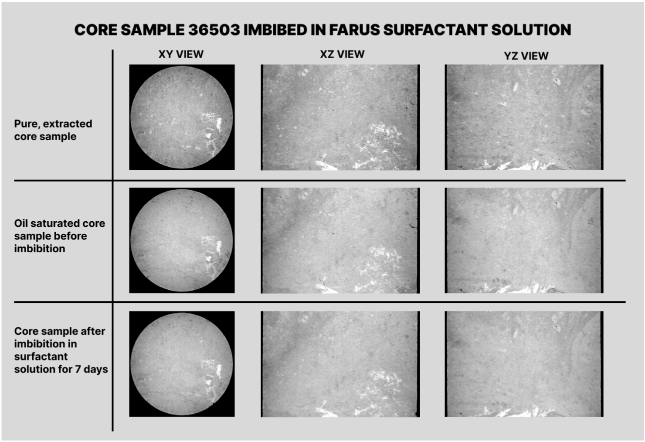

(a) Imbibition with non-ionic surfactant

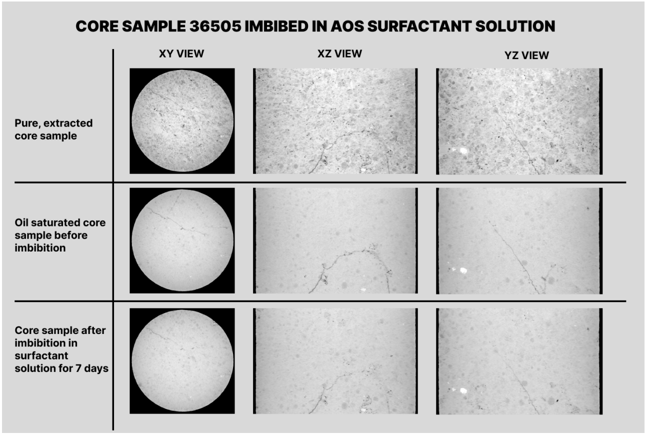

(b) Imbibition with anionic surfactant

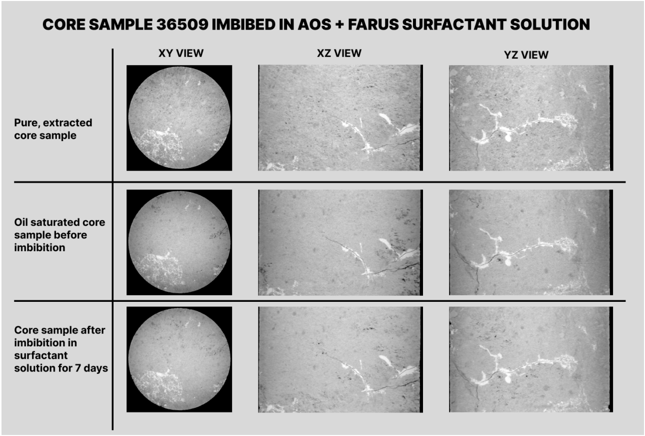

(c) Imbibition with anionic-nonionic surfactant

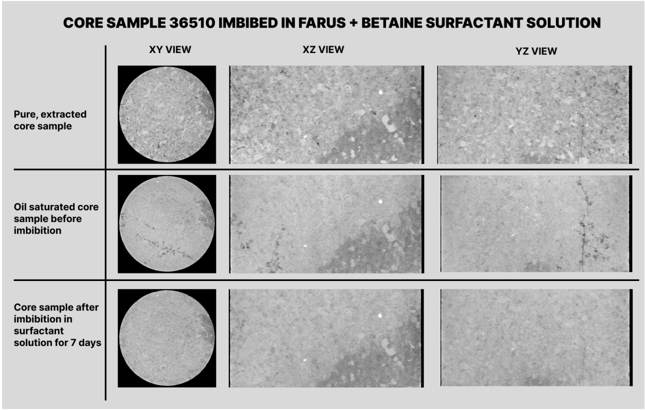

(d) Imbibition with zwitterionic-nonionic surfactant

Fig. S2: Computed tomography scan images of core samples before and after spontaneous imbibition experiments

The figures may not provide an immediate visual depiction of oil-surfactant interactions, but they reveal critical information about the structural changes and fluid distributions within the core samples. Notably, the presence of fractures cutting through the core samples along their diameters is indicative of the inherent heterogeneity of the rocks. These fractures serve as pathways for fluid flow during the imbibition process. Moreover, the images show variations in density within the core samples, which can be attributed to the heterogeneous nature of the rock, containing minerals, clay particles, or other solid components. These variations in density can affect fluid distribution and flow patterns within the porous rock.

A crucial observation in these images is the rough texture of the matrix of pure, extracted core samples. This roughness is primarily due to the absence of any fluid within the pores. This is a critical reference point for evaluating the success of the imbibition process. In contrast, the CT scan images of the oil-saturated core samples and those after surfactant imbibition show a much smoother texture. The smoothness is a direct result of the majority of pores being filled with either oil or the surfactant solution. As the fluids enter the pores, they replace the air or gas phase, resulting in a smoother appearance. This transformation in texture highlights the efficient penetration of fluids into the core material during the imbibition process.
